# Prognostic Value of Tumor-Infiltrating Lymphocytes and Tertiary Lymphoid Structures in Epstein-Barr Virus-Associated and -Negative Gastric Carcinoma

**DOI:** 10.3389/fimmu.2021.692859

**Published:** 2021-07-01

**Authors:** Na Cheng, Peng Li, Huanhuan Cheng, Xiaoxiao Zhao, Min Dong, Yiwang Zhang, Peizhen Zhao, Jianning Chen, Chunkui Shao

**Affiliations:** ^1^ Department of Pathology, The Third Affiliated Hospital, Sun Yat-Sen University, Guangzhou, China; ^2^ Department of Histology and Embryology of Basic Medical Department, Guangdong Medical University, Dongguan, China; ^3^ Department of Ophthalmology, The Third Affiliated Hospital, Sun Yat-Sen University, Guangzhou, China; ^4^ Department of Pathology, The Central Hospital of Wuhan, Huazhong University of Science and Technology, Wuhan, China; ^5^ Department of Medical Oncology, The Third Affiliated Hospital, Sun Yat-Sen University, Guangzhou, China; ^6^ Dermatology Hospital, Southern Medical University, Guangzhou, China

**Keywords:** tumor-infiltrating lymphocytes, tertiary lymphoid structures, EBV-associated gastric carcinoma, EBV-negative gastric carcinoma, nomogram, prognosis

## Abstract

**Background:**

Tumor-infiltrating lymphocytes (TILs) are considered a manifestation of the host immune response against cancer and tertiary lymphoid structures (TLS) may contribute to lymphocytes recruitment. Both of them have been reported as potential prognostic parameters in some human malignancies. However, the roles of TILs, TLS, and their correlation in Epstein-Barr Virus-associated gastric carcinoma (EBVaGC) and EBV-negative gastric carcinoma (EBVnGC) are largely unknown.

**Methods:**

To observe the correlation among TILs, TLS, and clinicopathological characteristics and their prognostic significance in EBVaGC and EBVnGC, respectively. TILs and TLS were assessed by morphology and/or immunohistochemistry, and accompanied by clinicopathological analysis from 846 gastric cancer patients in multiple institutions.

**Results:**

Forty-two (5.0%) cases of EBVaGC and 804 cases of EBVnGC were identified by *in situ* hybridization, respectively. For EBVnGC, higher TILs grade was correlated with TLS-present. EBVnGC patients with high TILs grade and TLS-present exhibited survival benefits. TILs (*P* = 0.001) and TLS (*P* = 0.003), especially TILs & TLS (*P* < 0.001) were independent prognostic factors in EBVnGC. A nomogram was constructed and validated for predicting the probability of overall survival and performed well with a good calibration. No significant prognostic value was detected in EBVaGC.

**Conclusion:**

TILs and TLS, especially TILs & TLS were promising prognostic indicators for overall survival in EBVnGC. TILs and TLS were highly overlapping in their extent and prognostic abilities, and may be considered as a coindicator of prognosis of gastric cancer. The evaluations of TILs and TLS are simple and can be assessed routinely in pathological diagnosis.

## Introduction

Gastric carcinoma (GC) is the fourth leading cause of cancer-related mortality worldwide and the most prevalent cancer in Eastern Asia (1, 2). Immunity plays a key role in tumor initiation and progression, with immune modulation considered to be an important strategy for cancer therapy. As the major type of infiltrating immune cells, tumor-infiltrating lymphocytes (TILs) are a heterogeneous group containing T cells, B cells, and natural killer cells, which have been reported to be related to favorable prognosis in various tumors such as melanoma, breast and nasopharyngeal carcinomas (3–5). The low TILs density could predict regional lymph node metastasis and poor prognosis for recurrence free survival in GC (6). Some suggested that TILs may direct patient selection for immune checkpoint blockade therapy in GC (7, 8). However, a large proportion of patients do not respond to immunotherapy, suggesting other possible immune factors may play a certain role in tumor microenvironment (9, 10).

Tertiary lymphoid structures (TLS), characterized by ectopic aggregated lymphocytes with high endothelial venules, have gained attention because of its correlation with prolonged patient’s survival in some tumors (11, 12). The formation and regulation of TLS involve the same chemokines and cytokines networks that orchestrate lymphoid organogenesis (13, 14). TLS have been reported to be associated with lymphocyte infiltration, represent a privileged area to provide a pathway for the recruitment of TILs, and generate the central-memory T and B cells to limit cancer progression (15, 16). Meanwhile, TLS could cooperate with TILs in a coordinated antitumor immune response (17). The exact prognostic role and the relationship between TILs and TLS in GC remain largely unknown.

Additionally, the association between Epstein-Barr virus (EBV) and GC is thought to be a predictive indicator for immunotherapy (18). Compared with EBV-negative GC (EBVnGC), EBV-associated GC (EBVaGC) has distinct clinicopathological features and most exhibit histology rich in lymphocyte infiltration and relatively favorable prognosis (19, 20).

The present study investigated TILs and TLS in the tumor tissues of patients with GC and evaluate their prognostic significance. In addition, the relationship between tumoral immune parameters such as TILs, TLS, TILs & TLS, and clinicopathological features in 42 EBVaGC and 804 EBVnGC patients was determined.

## Materials and Methods

### Patients and Specimens

Eight hundred forty-six cases of surgically resected GC were collected from multiple institutions including the First, Third, and Six Affiliated Hospitals of Sun Yat-sen University, from January 2001 to December 2013. An additional 86 GC patients from the Sun Yat-sen Memorial Hospital of Sun Yat-sen University (July 2008 to December 2011) were selected as a validation cohort for the nomogram. None of the patients underwent systematic chemotherapy or radiotherapy before surgery. Cases with cancer confined to mucosa were excluded because they have an excellent prognosis regardless of number of TILs.

Standard pathologic analyses were performed blindly by two experienced pathologists (CN, LP). Any discrepancy was reviewed to reach consensus at a multi-headed microscope. More than two H&E–stained section slides with tumor were obtained per case, and the mean number of slides was 4.72 (range, 3–14). In these slides, at least one slide contained the tumor invasive margin. Clinicopathological data were retrieved from the archives of the medical records and pathologic reports. All patients were restaged according to the American Joint Committee on Cancer (AJCC) Staging Manual, Seventh Edition (21).

Patients’ clinical outcomes were followed up from the date of GC resection until death or December 31, 2016. The data of patients who were alive at the last follow-up date and of those died from a cause other than GC were regarded as censored data.

This study was approved by the Institute Research Ethics Committees of the First, Third, Six Affiliated Hospitals and Sun Yat-sen Memorial Hospital, Sun Yat-sen University. All participants provided written informed consents prior to surgery.

### *In Situ* Hybridization for EBER-1

ISH assay was performed with an EBER-1 oligonucleotide probe (PanPath, Amsterdam, Netherlands), as previously described by Chen et al. (22). Dark brown nuclear staining was considered to be a positive signal. The known EBER-1-positive nasopharyngeal carcinoma tissues were used as the positive control and a sense probe for EBER-1 was used as the negative control.

### Immunohistochemistry

Immunohistochemical staining was performed on 4-µm thick sections of tissue samples using an automatic staining device (Ventana Benchmark Ultra immunostainer, Ventana Medical Systems, Inc., Tucson, USA). Antibodies were as follows: mouse anti-CD3 (clone LN10, 1:100, Novocastra), mouse anti-CD20 (clone L26, 1:250, Novocastra), and mouse anti-CD21 (clone 2G9, prediluted, Novocastra). PBS was used as the negative control. A cervical lymph node served as the positive control.

### Evaluation of TILs and TLS

No current consensus exists on the morphologic evaluation of TILs in GC, so we adopted and modified the TIL scoring recommendation used in previous studies (23–25). Briefly, global TILs are defined as the mean percentage of the invasive tumor area (including the tumor bed and peri-tumoral stroma) occupied by lymphocytes and plasma cells (23, 26), which was assessed by using a continuous scale as a semiquantitative parameter in 10% increments; if less than 10%, a 1 or 5% criteria was used. All available full-face tumor sections were evaluated, with no focus on hotspots. Area with necrosis, hemorrhage, or crush artifacts was excluded for TILs evaluation.

GC with lymphoid stroma, a rare histological variant of GC with prominent lymphocytic infiltration into the tumor and surrounding stroma, has distinctive clinicopathological and molecular features and is associated with a significantly better prognosis (24, 27). Therefore, patients with TILs level of >50% were classified as a separate group, and patients with TILs level of ≤50% were subdivided into two categories based on the mean value, which was determined as a threshold for survival analysis. As a whole, TILs were divided into three groups: grade 1 (minimal, ≤10%), grade 2 (moderate, 10–50%), and grade 3 (abundant, >50%).

All available sections were screened for the presence of TLS. First, the presence of lymphoid aggregates (LAs) was confirmed, as well as their patterns of organization at the tumor invasive margin and/or within the stroma of GC. Second, LAs with the visible germinal centers were considered as TLS. Third, LAs without visible germinal center were selectively stained by immunohistochemistry. The well-organized LAs with one or more CD20^+^ B cells aggregations containing CD21^+^ FDCs, surrounded by a CD3^+^ T cells rich area were defined as TLS. LAs in the mucosa or submucosa of stomach were excluded (28).

### Statistical Analysis

Comparisons among clinicopathologic features, EBV status, TILs, and TLS were performed by the Pearson Chi-Square test or Fisher’s exact test. Pearson correlation analysis was used to examine the correlation between TILs and TLS. Survival distribution was compared using the Kaplan-Meier method and the log-rank test. Prognostic variables associated with overall survival were examined by univariate analyses using a Cox proportional hazards regression model. Only those variables which were significantly associated with survival were enrolled into multivariate regression analyses. A nomogram was generated by R software 3.3, with the discriminative ability assessed by the concordance index (C-index), which ranges from 0.5 (no discrimination at all) to 1.0 (perfect discrimination). Calibration plots were generated to compare the predicted probability of overall survival with the observed outcome. Furthermore, the precision of survival predictions was evaluated using the area under receiver operating characteristic (ROC) curve (AUC) in the validation cohort. Two-sided *P* < 0.05 was considered statistically significant. Statistical analyses were performed using SPSS 17.0 statistics software (SPSS Inc., Chicago, IL, USA).

## Results

### Clinicopathological Features of EBVaGC and EBVnGC

**Figure 1:**
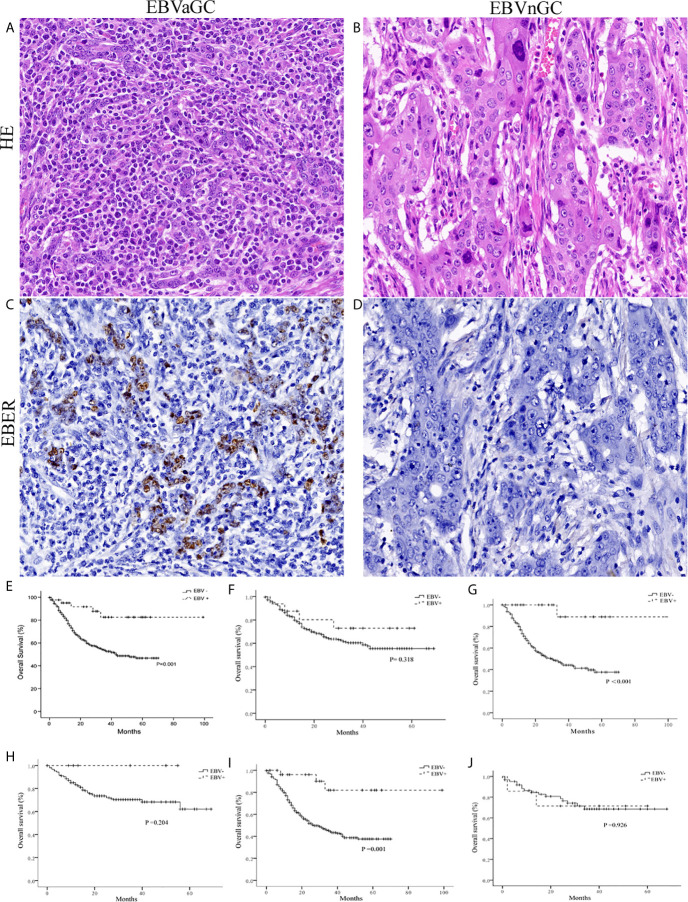
Histology, EBER-1 ISH, and survival curves in EBVaGC and EBVnGC. Histology of the representative cases of EBVaGC **(A)** and EBVnGC **(B)** (×400). EBER-1 ISH revealed strong nuclear staining in EBVaGC **(C)**, but not in EBVnGC **(D)** (×400). **(E)** Kaplan-Meier survival curves for overall survival in 42 cases of EBVaGC and 804 cases of EBVnGC. EBVaGC had better prognosis than EBVnGC (P = 0.001, Log-rank test). When patients were stratified based on tumor size [<5 cm **(F)**; >5 cm **(G)**] and Lauren classification [Intestinal **(H)**, Diffuse **(I)**, Mixed **(J)**], EBVaGC exhibited longer overall survival than EBVnGC in patients with tumor size >5 cm (P < 0.001) and Lauren diffuse type (P = 0.001).

According to the ISH results (**Figures 1A** **–D**), 42 of the 846 cases (5.0%) were identified as EBVaGC. As presented in **Table 1**, EBVaGC displayed distinct clinicopathological features, including younger age (*P* = 0.010), male predominance (*P* = 0.003), proximal stomach location (*P* = 0.006), bigger in tumor size (*P* = 0.039), Lauren diffuse type (*P* = 0.043), and higher grade of TILs (*P* < 0.001).

**Table 1:** Clinicopathological characteristics of EBVaGC and EBVnGC.

Characteristics	All cases	EBVaGC	EBVnGC	*P* value
	*n* (%)	*n* (%)	*n* (%)	
Total, *n*	846	42 (5.0)	804 (95.0)	
Age, y				0.010
<60	454 (53.7)	31 (73.8)	423 (52.6)	
≥60	392 (46.3)	11 (26.2)	381 (47.4)	
Mean ± SD	57.2 ± 12.7	53.2 ± 12.4	57.4 ± 12.6	
Gender				0.003
Male	585 (69.1)	38 (90.5)	547 (68.0)	
Female	261 (30.9)	4 (9.5)	257 (32.0)	
Location				0.006
Cardia, fundus	223 (26.4)	13 (31.0)	210 (26.1)	
Body	214 (25.3)	19 (45.2)	195 (24.3)	
Antrum	380 (44.9)	9 (21.4)	371 (46.1)	
Remnant/Multiple sites	29 (3.4)	1 (2.4)	28 (3.5)	
Size				0.039
<5 cm	457 (54.0)	16 (38.1)	441(54.9)	
≥5 cm	389 (46.0)	26 (61.9)	363 (45.1)	
pTNM stage*				0.339
I+II	375 (44.3)	22 (52.4)	353 (43.9)	
III+IV	471 (55.7)	20 (47.6)	451 (56.1)	
Lymphovascular invasion				0.138
Absent	701 (82.9)	31 (73.8)	670 (83.3)	
Present	145 (17.1)	11 (26.2)	134 (16.7)	
Perineural invasion				0.835
Absent	698 (82.5)	34 (81.0)	664 (82.6)	
Present	148 (17.5)	8 (19.0)	140 (17.4)	
Histologic differentiation				0.103
Well/Moderate	220 (26.0)	6 (14.3)	214 (26.6)	
Poor	626 (74.0)	36 (85.7)	590 (73.4)	
Lauren classification				0.043
Intestinal	221 (26.1)	6 (14.3)	215 (26.7)	
Diffuse	556 (65.7)	29 (69.0)	527 (65.5)	
Mixed	69 (8.2)	7 (16.7)	62 (7.7)	
WHO classification				0.073
Pap/tub	224 (26.5)	6 (14.3)	218 (27.1)	
Muc/por	622 (73.5)	36 (85.7)	586 (72.9)	
TILs				<0.001
Grade 1	683 (80.7)	22 (52.4)	661 (82.2)	
Grade 2	146 (17.3)	15 (35.7)	131 (16.3)	
Grade 3	17 (2.0)	5 (11.9)	12 (1.5)	
TLS				0.893
Absent	254 (30.0)	13 (31.0)	241 (30.0)	
Present	592 (70.0)	29 (69.0)	563 (70.0)	

EBVaGC, EBV-associated gastric carcinoma; EBVnGC, EBV-negative gastric carcinoma; por, poorly cohesive carcinoma; pap, papillary adenocarcinoma; tub, well and moderately differentiated tubular adenocarcinoma; muc, mucinous adenocarcinoma; TILs, tumor-infiltrating lymphocytes; TLS, tertiary lymphoid structures. *The 7th AJCC TNM staging system.

During a mean of 22.1 (range, 1–99) months of follow-up, 5 (12%) patients in EBVaGC and 309 (38%) ones in EBVnGC group died. Kaplan-Meier analysis revealed that patients of EBVaGC had significantly better overall survivals than that of EBVnGC (*P* = 0.001, **Figure 1E**). While stratified by tumor size and Lauren classification, EBVaGC exhibited better overall survivals than EBVnGC in patients with tumor size >5 cm (P < 0.001, **Figure 1G**) and Lauren diffuse type (P = 0.001, **Figure 1I**). No statistically significant difference was observed in EBVaGC and EBVnGC patients with tumor size <5 cm (**Figure 1F**), Lauren intestinal and mixed types (**Figures 1H, J**).

### Comparison of Clinicopathologic Characteristics According to TILs

**Figure 2:**
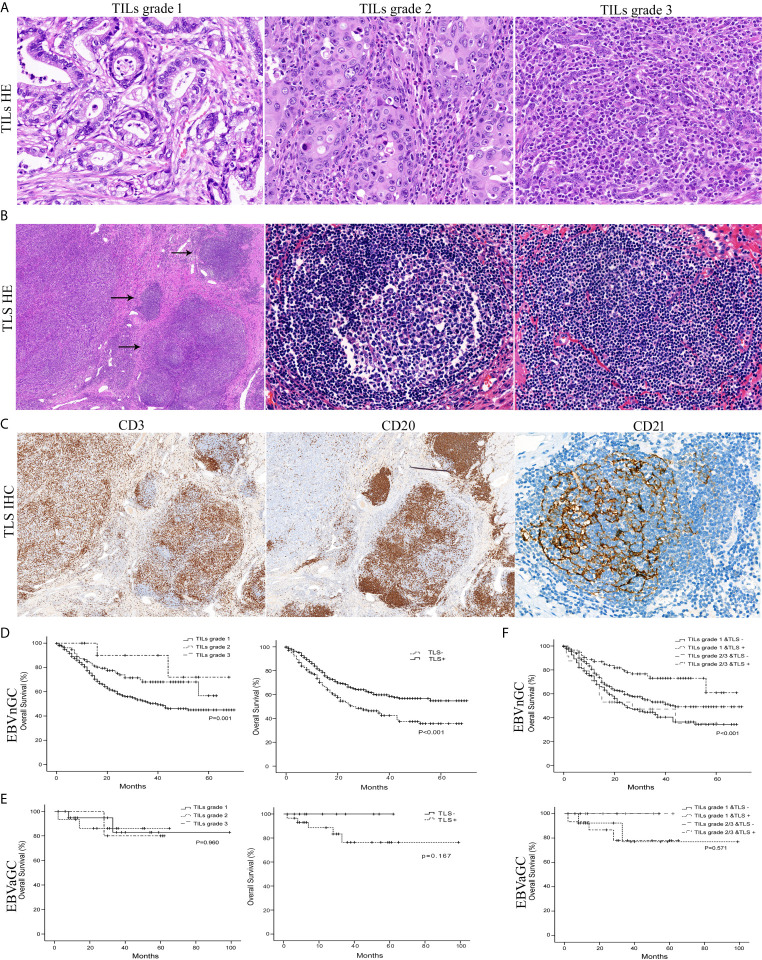
Histology of the TILs grade, TLS, and survival curves in EBVaGC and EBVnGC. **(A)** The mean percentage of the stromal area occupied by lymphocytes and plasma cells within tumor was assessed as TILs grade 1 (minimal, ≤10%), grade 2 (moderate, 10–50%), and grade 3 (abundant, >50%) (H&E, ×400). **(B)** TLS (arrows) with or without germinal centers (center) were mainly localized at the invasive margin of cancer (left field) (H&E, left ×50, center ×400, right ×400). **(C)** Whether germinal centers were visible or not, clusters of CD20^+^ B lymphocytes (×50) in TLS were surrounded by CD3^+^ T cell areas (×50) and contained a network of CD21^+^ FDCs (×400) by immunohistochemical staining. Kaplan-Meier survival analyses for overall survival were performed according to the TILs grade, TLS, or TILs & TLS in EBVnGC **(D, F)** top and EBVaGC **(E, F)** bottom.

To identify the clinicopathological significance of TILs, we divided the specimens into three groups (grade 1 TILs ≤10%, grade 2 TILs 10–50%, and grade 3 TILs >50%) (**Figure 2A**). For EBVnGC, a summary of the clinicopathological characteristics according to the grade of TILs is shown in **Table 2**. The tumor with higher grade of TILs was bigger in size (*P* = 0.028). According to Lauren classification, there was a significant association between the TILs density and the diffuse/mixed type (*P* < 0.001).

**Table 2:** Correlation of the TILs grade and TLS with clinicopathological characteristics in EBVnGC.

Characteristics	All cases *n* (%)	TILs grade			*P* value	TLS		*P* value
		1	2	3		Absent	Present	
Total, *n*	804	661 (82.2)	131 (16.3)	12 (1.5)		241 (28.5)	563 (66.5)	
Age, y					0.089			0.010
<60	423 (52.6)	356 (53.9)	64 (48.9)	3 (25.0)		110 (45.6)	313 (55.6)	
≥60	381 (47.4)	305 (46.1)	67 (51.1)	9 (75.0)		131 (54.4)	250 (44.4)	
Mean ± SD	57.4 ± 12.6	57.0 ± 12.7	58.5 ± 12.1	64.3 ± 14.1		59.1 ± 12.9	56.7 ± 12.5	
Gender					0.100			0.411
Male	547 (68.0)	441 (66.7)	99 (75.6)	7 (58.3)		169 (70.1)	378 (67.1)	
Female	257 (32.0)	220 (33.3)	32 (24.4)	5 (41.7)		72 (29.9)	185 (32.9)	
Location					0.505			0.021
Cardia, fundus	210 (26.1)	173 (26.2)	32 (24.4)	5 (41.7)		60 (24.9)	150 (26.6)	
Body	195 (24.3)	164 (24.8)	27 (20.6)	4 (33.3)		50 (20.7)	145(25.8)	
Antrum	371 (46.1)	300 (45.4)	68 (51.9)	3 (25.0)		116 (48.1)	255 (45.3)	
Remnant/Multiple sites	28 (3.5)	24 (3.6)	4 (3.1)	0		15 (6.2)	13 (2.3)	
Size					0.028			0.013
<5 cm	441 (54.9)	366 (55.4)	73 (55.7)	2 (16.7)		116 (48.1)	325 (57.7)	
≥5 cm	363 (45.1)	295 (44.6)	58 (44.3)	10 (83.3)		125 (51.9)	238 (42.3)	
pTNM stage*					0.483			0.036
I+II	353 (43.9)	285 (43.1)	61 (46.6)	7 (43.9)		92 (38.2)	261 (46.4)	
III+IV	451 (56.1)	376 (56.9)	70 (53.4)	5 (41.7)		149 (61.8)	302 (53.6)	
Lymphovascular invasion					0.858			0.217
Absent	670 (83.3)	548 (82.9)	112 (85.5)	10 (83.3)		207 (85.9)	463 (82.2)	
Present	134 (16.7)	113 (17.1)	19 (14.5)	2 (16.7)		34 (14.1)	100 (17.8)	
Perineural invasion					0.051			0.761
Absent	664 (82.6)	537 (81.2)	115 (87.8)	12 (100)		201 (83.4)	463 (82.2)	
Present	140 (17.4)	124 (18.8)	16 (12.2)	0		40 (16.6)	100 (17.8)	
Histologic differentiation					0.411			0.007
Well/Moderate	214 (26.6)	182 (27.5)	30 (22.9)	2 (16.7)		80 (33.2)	134 (23.8)	
Poor	590 (73.4)	479 (72.5)	101(77.1)	10 (83.3)		161 (66.8)	429 (76.2)	
Lauren classification					<0.001			0.009
Intestinal	215 (26.7)	184 (27.8)	30 (22.9)	1 (8.3)		80 (33.2)	135 (24.0)	
Diffuse	527 (65.5)	441 (66.7)	78 (59.5)	8 (66.7)		149 (61.8)	378 (67.1)	
Mixed	62 (7.7)	36 (5.4)	23 (17.6)	3 (25.0)		12 (5.0)	50 (8.9)	
WHO classification					0.424			0.007
Pap/tub	218 (27.1)	185 (28.0)	31 (23.7)	2 (16.7)		81 (33.6)	137 (24.3)	
Muc/por	586 (72.9)	476 (72.0)	100 (76.3)	10 (83.3)		160 (66.4)	426 (75.7)	

EBVnGC, EBV-negative gastric carcinoma; por, poorly cohesive carcinoma; pap, papillary adenocarcinoma; tub, well and moderately differentiated tubular adenocarcinoma; muc, mucinous adenocarcinoma; TILs, tumor-infiltrating lymphocytes; TLS, tertiary lymphoid structures. *The 7th AJCC TNM staging system.

For EBVaGC, no statistically significant difference was observed, except for gender. The patients with increasing TILs density were more likely to be male (*P* = 0.046; **Supplementary Table 1**). The proportion of TILs grade 2 and 3 in EBVaGC is 47.6%, significantly higher than that in EBVnGC (17.8%) (P < 0.001; **Table 1**).

### Comparison of Clinicopathologic Characteristics According to TLS

TLS are highly organized structures with or without germinal center (**Figures 2B** **, C**). Among the total 804 EBVnGC patients, 563 (66.5%) cases showed the presence of TLS. Patients with the presence of TLS were younger age (*P* = 0.010), smaller in tumor size (*P* = 0.013), high pTNM stage (*P* = 0.036), poorly histologic differentiation (*P* = 0.007), Lauren diffuse type (*P* = 0.009), and WHO poorly differentiated type (*P =* 0.007) (**Table 2**).

For EBVaGC, no statistically significant difference was observed (**Supplementary Table 1**). However, the proportion of TLS-present patients was higher than that of TLS-absent ones (69.0% and 31.0%, respectively) (**Supplementary Table 1**).

### Association Between TILs and TLS in EBVaGC and EBVnGC

The presence of TLS in EBVnGC was related with TILs (*P* = 0.001; **Table 3**). The proportion of TILs grade 2 and 3 in TLS-present patients was 21.2%, obviously higher than that in TLS-absent ones (9.9%) (**Table 3**). However, there was no significant association between TILs and TLS in EBVaGC (**Table 3**).

**Table 3:** Association between TILs grade and TLS in EBVnGC and EBVaGC.

Variables	EBVnGC			*P* value	EBVaGC			*P* value
	*n* (%)	TLS-absent	TLS-present		*n* (%)	TLS-absent	TLS-present	
TILs				0.001				0.701
grade 1	661 (82.2)	217 (32.8)	444 (67.2)		22 (52.4)	8 (61.5)	14 (48.3)	
grade 2	131 (16.3)	22 (16.8)	109 (83.2)		15 (35.7)	4 (30.8)	11 (37.9)	
grade 3	12 (1.5)	2 (16.7)	10 (83.3)		5 (11.9)	1 (7.7)	4 (13.8)	
Total, *n*	804 (100.0)	241 (30.0)	563 (70.0)		42 (100.0)	13 (31.0)	29 (69.0)	

TILs, tumor-infiltrating lymphocytes; TLS, tertiary lymphoid structures.

### Prognostic Significance of TILs and TLS in EBVaGC and EBVnGC

We detected that EBVnGC patients with higher TILs grade and the presence of TLS showed survival benefits according to Kaplan-Meier survival analysis (**Figure 2D**). No significant prognostic value was detected in EBVaGC (**Figure 2E**).

In the univariate analysis of EBVnGC, the clinical parameters of tumor location, size, pTNM stage, lymphovascular invasion, perineural invasion, histologic differentiation, Lauren classification, WHO classification, TILs, and TLS were found to be significantly associated with overall survival (**Table 4**). The multivariate model revealed that pTNM stage (HR 5.025; 95% CI 3.745–6.743; *P* < 0.001), lymphovascular invasion (HR 2.053, 95% CI 1.571–2.684, *P* < 0.001), perineural invasion (HR 1.649, 95% CI 1.267–2.146, *P* < 0.001), histologic differentiation (HR 1.817, 95% CI 1.347–2.400, *P* < 0.001), Lauren classification (HR 1.782, 95% CI 1.323–2.662, *P* < 0.001), WHO classification (HR 1.798, 95% CI 1.337–2.416, *P* < 0.001), TILs (HR 1.830, 95% CI 1.295–2.586, *P* = 0.001), and TLS (HR 1.558, 95% CI 1.228–1.977, *P* = 0.003) were independent prognostic factors for overall survival (**Table 4**).

**Table 4:** Cox proportional hazards regression models for the predictors of overall survival in EBVnGC.

Variables	Categories	Univariate analysis		Multivariate analysis	
		HR (95% CI)	*P* value	HR (95% CI)	*P* value
Age (y)	≥ 60 vs < 60	1.245 (0.988–1.568)	0.063		
Gender	female *vs* male	0.973 (0.761–1.244)	0.828		
Location	body *vs* cardia/fundus	0.945 (0.680–1.312)	0.735		
	antrum *vs* cardia/fundus	0.932 (0.701–1.238)	0.627		
	Remnant/multiple sites *vs* cardia/fundus	3.081 (1.859–5.106)	<0.001		
Size	≥5 cm *vs <*5 cm	1.419 (1.126–1.788)	0.003		
pTNM stage*	III+IV *vs* I+II	4.991 (3.722–6.694)	<0.001	5.025 (3.745–6.743)	<0.001
Lymphovascular invasion	present *vs* absent	2.120 (1.628–2.760)	<0.001	2.053 (1.571–2.684)	<0.001
Perineural invasion	present *vs* absent	1.771 (1.366–2.297)	<0.001	1.649 (1.267–2.146)	<0.001
Histologic differentiation	poor *vs* well/moderate	1.777 (1.322–2.389)	<0.001	1.817 (1.347–2.400)	<0.001
Lauren classification	diffuse/mixed *vs* intestinal	1.744 (1.300–2.340)	<0.001	1.782 (1.323–2.662)	<0.001
WHO classification	muc/por *vs* pap/tub	1.758 (1.313–2.354)	<0.001	1.798 (1.337–2.416)	<0.001
TILs	grade 1 *vs* grade 2/3	1.846 (1.306–2.607)	0.001	1.830 (1.295–2.586)	0.001
TLS	absent *vs* present	1.647 (1.300–2.085)	<0.001	1.558 (1.228–1.977)	0.003
TILs & TLS	grade 1&TLS− *vs* grade 2/3&TLS+	2.844 (1.864–4.340)	<0.001	2.683 (1.756–4.099)	<0.001
	grade 1&TLS+ *vs* grade 2/3&TLS+	2.039 (1.356–3.066)	0.001	2.005 (1.332–3.019)	0.001
	grade 2/3&TLS− *vs* grade 2/3&TLS+	2.879 (1.485–5.581)	0.002	2.411 (1.233–4.715)	0.010

EBVnGC, EBV-negative gastric carcinoma; por, poorly cohesive carcinoma; pap, papillary adenocarcinoma; tub, well and moderately differentiated tubular adenocarcinoma; muc, mucinous adenocarcinoma; TILs, tumor-infiltrating lymphocytes; TLS, tertiary lymphoid structures; HR, hazard ratio; CI, confidence interval. *The 7th AJCC TNM staging system.

For EBVnGC, even though TILs and TLS have a certain correlation (r = 0.139, *P* < 0.001), some tumors with moderate to abundant TILs did not show the presence of TLS. Therefore, we divided tumors into four groups according to TILs (grade 1 *vs* grade 2/3) and TLS (absent or present). As shown in **Figure 2F**, patients with higher grades of TILs and the presence of TLS had the significantly best overall survival than the other three groups. The univariate and multivariate analysis also confirmed that TILs and TLS was significantly and independently associated with better survival.

### Prognostic Nomogram in EBVnGC and Validation of Predictive Accuracy of the Nomogram for Overall Survival

**Figure 3:**
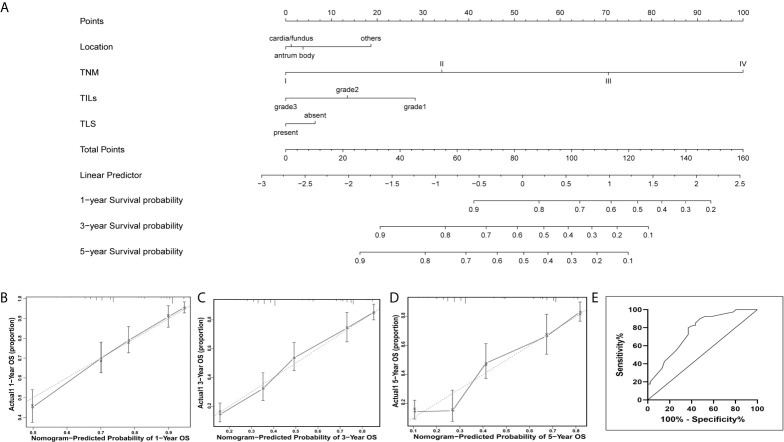
Nomogram for predicting prognosis in patients with EBVnGC. **(A)** A predictive nomogram for 1-year, 3-year, and 5-year overall survival was generated by combining significant independent prognostic factors including location, TNM stage, TILs, and TLS. To estimate the survival in a given patient, the “Total Points” score is calculated by summing the respective “Points” values corresponding to each variable. Using this “Total Points” score, the survival probabilities at 1, 3, and 5 years can be predicted according to the lower scales. Calibration plots of the nomogram for 1-year **(B)**, 3-years **(C)**, and 5-years **(D)** overall survival. Dotted Line, ideal model; vertical bars, 95% confident interval. **(E)** Predictive accuracy of the nomogram for overall survival were confirmed in the external validation cohort, indicating the model was reliable.

A prognostic nomogram was depicted to predict 1-, 3-, and 5-year individualized absolute risk for mortality based on significant factors among all EBVnGC patients (**Figure 3A**). Significant attributes were selected by the multivariate stepwise regression analysis, including location, pTNM stage, TILs, and TLS (all *P* < 0.05). Predictive accuracy of the nomogram was good, with the C-index being 0.751 (95% CI 0.724–0.779). Calibration curves for 1-, 3-, and 5-year survival prediction indicated good agreement between predicted probabilities and actual observations (**Figures 3B** **–D**).

For the external validation cohort, the mean follow-up time was 32.1 months (range 1–82 months). A summary of clinicopathological characteristics was shown in **Supplementary Table 2**. Predictive accuracy of the nomogram for overall survival was good, with the AUC value of 0.759 (95% CI, 0.641 to 0.848), indicating the nomogram was useful for predicting survival of patients with GC (**Figure 3E**).

## Discussion

In this study, TILs and TLS, especially TILs & TLS correlated with the clinical outcome of GC. Patients with higher TILs grade and TLS-present exhibited survival benefits in EBVnGC. TILs were associated with TLS and both were promising independent prognostic factors of EBVnGC. Moreover, we established a nomogram model that combined the TILs grade and TLS status as prognostic variables with other well-established prognostic factors in EBVnGC and found that the nomogram performed well for both calibration and external validation. The model may be the crucial determinants of clinical care for individual GC patients.

TILs were assessed on H&E sections and divided into three groups. EBVnGC patients with high TILs density showed markedly improved survival. The TILs grade was proved to be a promising independent prognostic indicator for overall survival in EBVnGC, which was in accordance with previous literature with regard to GC (23, 29) and other types of cancers (30–32). Generally, the predominance of TILs has been claimed to reflect an effective anti-tumor immune response, which was promoted by a dynamic and complex interaction between infiltrating immune cells and tumor cells, and this interaction is critical for tumor progression and clinical outcome (33, 34).

We found that the presence of TLS was a good, independent prognostic parameter for overall survival in EBVnGC. Despite heterogeneity in TLS-signatures and TLS-quantifying methods, most studies have consistently found the association between TLS and prolonged patients’ survival, suggesting the occurrence of an active immune response within TLS to tumor microenvironment (35, 36). Conversely, limited studies have detected that the presence of TLS was a negative prognostic factor and associated with more advanced disease in colorectal, breast, and hepatocellular carcinomas (37–39). The possible reason for this discrepancy was that the maintenance and function of TLS dictated by their cellular composition and the surrounding immune contexture may vary in different tumors (36).

In our study, 42 (5.0%) patients were identified as EBVaGC, with distinct clinicopathological features and significantly better prognosis. The high TILs density and the presence of TLS may be the possible reasons. Kang et al. assessed the prognostic value of TILs amongst EBVaGC and found that the TILs density was an independent predictor for recurrence free survival (6). However, in our study, limited numbers of EBVaGC patients and the uneven distribution of cases within each TILs and TLS group made little internal difference, so no significant prognostic value was found in EBVaGC. Further large-scale validation studies remain to be done to fully understand the exact prognostic role of TILs and TLS in EBVaGC.

Of note, TLS and the TILs were highly overlapping in their extent and prognostic abilities. Combination of the two has prognostic power superior to each one individually. Comparing to patients with high TILs grade but the absence of TLS, the ones with high TILs grade and the presence of TLS showed improved survival, suggesting that TLS may actively license the prognostic value of TILs. Dendritic cells or plasma cells expressing markers of antigen-specific responses within TLS were reported to be associated with increased responses of TILs, which propose that TLS may educate TILs to control tumors better (15, 40). Some studies demonstrated that TLS were correlated with TILs, contributing to TILs recruitment and cooperating with TILs in antitumor immune response in colorectal cancer (17) and breast cancer (41). Hennequin et al. found a significant correlation between the density of B cell aggregates and Tbet^+^ effector T cells in GC, which was also associated with better relapse-free survival, indicating that GC could be sustained through a complex network of tumor-infiltrating immune cells organized in TLS, allowing T/B cells coordination (42). The adhesion molecules, chemokines, and integrins may mediate migration of tumor-specific T cells into TLS. Meanwhile, TLS-serving HEVs may provide a gateway for the recruitment of circulating T lymphocytes into the tumor (43, 44).

Interestingly, we found a certain correlation between TILs and TLS, but some patients with moderate to abundant TILs did not develop TLS. The local tumor microenvironment including a series of signals or cytokines following the local cross-talk between TILs and resident stromal cells, may provide specific cues conducive to the formation of TLS (45, 46). We previously showed that CD3^+^ and CD8^+^ T lymphocytes as the predominant constituent cells of TILs in gastric cancer were associated with good prognosis (47), whereas tumor-infiltrating B cells especially when present in TLS, may be key players in anti-tumor immunity (48). Over half but not all diffuse type/genome stable GCs had enrichment of intratumoral TLS and exhibited different chemokine gene expression signature, reflecting signs of an initiated antitumor immune response and the different stages of lymphoid neogenesis (49). The presence of TLS may represent a privileged site where specific naïve B cells can undergo their final differentiation into effector B cells, such as memory B cells (48, 50). These suggest that TILs and TLS may interact with each other and play different roles in different stages of the anti-tumor immune response.

In conclusion, the present study indicated that high grade of TILs was associated with the presence of TLS and further elucidated that TILs and TLS, especially TILs & TLS were promising independent prognostic factors for overall survival in GC. TILs and TLS were highly overlapping in their extent and prognostic abilities, and could be considered as a coindicator of prognosis of gastric cancer. The evaluations of TILs and TLS are simple and can be assessed routinely in pathological diagnosis. TILs and TLS appear likely to be part of an adaptive immune response and may be helpful for understanding the immunobiology of the tumor microenvironment of gastric cancer.

## Data Availability Statement

The original contributions presented in the study are included in the article/**Supplementary Material**. Further inquiries can be directed to the corresponding authors.

## Ethics Statement

The studies involving human participants were reviewed and approved by the Institute Research Ethics Committees of the First, Third, and Six Affiliated Hospital and Sun Yat-Sen Memorial Hospital, Sun Yat-Sen University. The patients/participants provided their written informed consent to participate in this study.

## Author Contributions

CS and JC prepared the study concept and design. NC, PL, JC, and CS wrote the paper. NC and PL performed standard pathologic analysis. XZ, MD, and YZ collected the cohort data and samples. HC and PZ did data analysis and interpretation. CS and JC supervised the project. All authors contributed to the article and approved the submitted version.

## Funding

This work was supported by the National Natural Science Foundation of China (82073397), the Guangdong Basic and Applied Basic Research Foundation (2019A1515011455), the Natural Science Foundation of Guangdong Province (2018A030313650), the Guangzhou Science and Technology Project (202102010156, 202102010267), and the NSFC cultivating grant of The Third Affiliated Hospital, Sun Yat-sen University (2020GZRPYMS01, 2021GZRPYQN12), Guangdong Province, China.

## Conflict of Interest

The authors declare that the research was conducted in the absence of any commercial or financial relationships that could be construed as a potential conflict of interest.

## Abbreviations

TILs, tumor-infiltrating lymphocytes; TLS, tertiary lymphoid structures; GC, gastric carcinoma; EBVaGC, Epstein-Barr Virus-associated gastric carcinoma; EBVnGC, EBV-negative gastric carcinoma; ISH, *in situ* hybridization; EBER-1, EBV-encoded small RNA 1; H&E, hematoxylin and eosin; FDCs, follicular dendritic cells; AJCC, the American Joint Committee on Cancer; LAs, lymphoid aggregates.
