# Supplementary material for: Prognostic Value of Tumor-Infiltrating Lymphocytes and Tertiary Lymphoid Structures in Epstein-Barr Virus-Associated and -Negative Gastric Carcinoma
Source: Front Immunol. 2021 Jul 1;12:692859. doi: 10.3389/fimmu.2021.692859 (PMC8281029; doi:10.3389/fimmu.2021.692859)
Supplement: Supplementary file 1 [file DataSheet_1.docx]

**Supplementary Table 1** **Correlation of the TILs grade and TLS with clinicopathological characteristics in EBVaGC**

| **Characteristics** | **All cases**  ***n* (%)** | **TILs grade** | | | ***P* value** | **TLS** | | ***P* value** |
| --- | --- | --- | --- | --- | --- | --- | --- | --- |
|  |  | **1** | **2** | **3** |  | **Absent** | **Present** |  |
| Total, *n* | 42 | 22 (52.4) | 15 (35.7) | 5 (11.9) |  | 13 (31.0) | 29 (69.0) |  |
| Age, y |  |  |  |  | 0.881 |  |  | 0.270 |
| < 60 | 31 (73.8) | 17 (77.3) | 11 (73.3) | 3 (60.0) |  | 8 (61.5) | 23 (79.3) |  |
| ≥ 60 | 11 (26.2) | 5 (22.7) | 4 (26.7) | 2 (40.0) |  | 5 (38.5) | 6 (20.7 ) |  |
| Mean ± SD | 53.2 ±12.4 | 50.7 ±13.1 | 55.7 ±10.3 | 56.6 ±15.5 |  | 53.9±11.8 | 52.9±12.9 |  |
| Gender |  |  |  |  | 0.046 |  |  | 0.787 |
| Male | 38 (90.5) | 20 (90.9) | 15 (100) | 3 (60.0) |  | 12 (92.3) | 26 (89.7) |  |
| Female | 4 (9.5) | 2 (9.1) | 0 | 2 (40.0) |  | 1 (7.7) | 3 (10.3) |  |
| Location |  |  |  |  | 0.904 |  |  | 0.246 |
| Cardia, fundus | 13 (31.0) | 8 (36.4) | 4 (26.7) | 1 (20.0) |  | 4 (30.8) | 9 (31.0) |  |
| Body | 19 (45.2) | 9 (40.9) | 8 (53.3) | 2 (40.0) |  | 7 (53.8) | 12 (41.4) |  |
| Antrum | 9 (21.4) | 4 (18.2) | 3 (20.0) | 2 (40.0) |  | 1 (7.7) | 8 (27.6) |  |
| Remnant/ Multiple sites | 1 (2.4) | 1 (4.5) | 0 | 0 |  | 1 (2.4) | 0 (0) |  |
| Size |  |  |  |  | 0.443 |  |  | 0.974 |
| <5 cm | 16 (38.1) | 7 (31.8) | 6 (40.0) | 3 (60.0) |  | 5 (38.5) | 11 (37.9) |  |
| ≥5 cm | 26 (61.9) | 15 (68.2) | 9 (60.0) | 2 (40.0) |  | 8 (61.5) | 18 (62.1) |  |
| pTNM stage* |  |  |  |  | 1.000 |  |  | 0.514 |
| I+II | 22 (52.4) | 11 (50.0) | 8 (53.3) | 3 (60.0) |  | 8 (61.5) | 14 (48.3) |  |
| III+IV | 20 (47.6) | 11 (50.0) | 7 (46.7) | 2 (40.0) |  | 5 (38.5) | 15 (51.7) |  |
| Lymphovascular invasion |  |  |  |  | 0.119 |  |  | 0.713 |
| Absent | 31 (73.8) | 16 (72.7) | 13 (86.7) | 2 (40.0) |  | 9 (69.2) | 22 (75.9) |  |
| Present | 11 (26.2) | 6 (27.3) | 2 (13.3) | 3 (60.0) |  | 4 (30.8) | 7 (24.1) |  |
| Perineural invasion |  |  |  |  | 0.859 |  |  | 0.398 |
| Absent | 34 (81.0) | 17 (77.3) | 13 (86.7) | 4 (80.0) |  | 12 (92.3) | 22 (75.9) |  |
| Present | 8 (19.0) | 5 (22.7) | 2 (13.3) | 1 (20.0) |  | 1 (7.7) | 7 (24.1) |  |
| Histologic differentiation |  |  |  |  |  |  |  | 0.353 |
| Well/ Moderate | 6 (14.3) | 4 (18.2) | 2 (13.3) | 0 | 0.746 | 3 (23.1) | 3 (10.3) |  |
| Poor | 36 (85.7) | 18 (81.8) | 13 (86.7) | 5 (100) |  | 10 (76.9) | 26 (89.7) |  |
| Lauren classification |  |  |  |  | 0.175 |  |  | 0.454 |
| Intestinal | 6 (14.3) | 4 (18.2) | 2 (13.3) | 0 |  | 3 (23.1) | 3 (10.3) |  |
| Diffuse | 29 (69.0) | 17 (77.3) | 8 (53.3) | 4 (80.0) |  | 9 (69.2) | 20 (69.0) |  |
| Mixed | 7 (16.7) | 1 (4.5) | 5 (33.3) | 1 (20.0) |  | 1 (7.7) | 6 (20.7) |  |
| WHO classification |  |  |  |  | 0.746 |  |  | 0.353 |
| Pap/ tub | 6 (14.3) | 4 (18.2) | 2 (13.3) | 0 |  | 3 (23.1) | 3 (10.3) |  |
| Muc/ por | 36 (85.7) | 18 (81.8) | 13 (86.7) | 5 (100) |  | 10 (76.9) | 26 (89.7) |  |

Abbreviations: EBVaGC, EBV-associated gastric carcinoma; por, poorly cohesive carcinoma; pap, papillary adenocarcinoma; tub, well and moderately differentiated tubular adenocarcinoma; muc, mucinous adenocarcinoma; TILs, tumour-infiltrating lymphocytes; TLS, tertiary lymphoid structures. * The 7th AJCC TNM staging system.

**Supplementary Table 2** The demographic and clinicopathological variables of the validation cohort

| **Variables** | **No. of Patients (%)** |
| --- | --- |
| Age, y |  |
| < 60 | 48 (55.8) |
| ≥ 60 | 38 (44.2) |
| Mean ± SD | 57.5 ± 13.9 |
| Gender |  |
| Male | 66 (76.7) |
| Female | 20 (23.3) |
| Location |  |
| Cardia, fundus | 19 (22.1) |
| Body | 30 (34.9) |
| Antrum | 34 (39.5) |
| Remnant/ Multiple sites | 3 (3.5) |
| Size |  |
| <5 cm | 42 (48.8) |
| ≥5 cm | 44 (51.2) |
| pTNM stage* |  |
| I+II | 32 (37.2) |
| III+IV | 54 (62.8) |
| Lymphovascular invasion |  |
| Absent | 63 (73.3) |
| Present | 23 (26.7) |
| Perineural invasion |  |
| Absent | 52 (60.5) |
| Present | 34 (39.5) |
| Histologic differentiation |  |
| Well/ Moderate | 29 (33.7) |
| Poor | 57 (66.3) |
| Lauren classification |  |
| Intestinal | 29 (33.7) |
| Diffuse | 47 (54.7) |
| Mixed | 10 (11.6) |
| WHO classification |  |
| Pap/ tub | 29 (33.7) |
| muc/ por | 57 (66.3) |
| TILs |  |
| grade 1 | 72 (83.7) |
| grade 2 | 13 (15.1) |
| grade 3 | 1 (1.2) |
| TLS |  |
| Absent | 66 (76.7) |
| Present | 20 (23.3) |

Abbreviations: por, poorly cohesive carcinoma; pap, papillary adenocarcinoma; tub, well and moderately differentiated tubular adenocarcinoma; muc, mucinous adenocarcinoma; TILs, tumour-infiltrating lymphocytes; TLS, tertiary lymphoid structures. * The 7th AJCC TNM staging system.
